# Supplementary material for: Novel Driver Strength Index highlights important cancer genes in TCGA PanCanAtlas patients
Source: PeerJ. 2022 Aug 11;10:e13860. doi: 10.7717/peerj.13860 (PMC9375969; doi:10.7717/peerj.13860)
Supplement: Supplemental Information 2 [file peerj-10-13860-s002.zip › COHORTS/genes plots/detailed/2021_8_16_14_9_distribution_events_detailed_males_15.pdf]

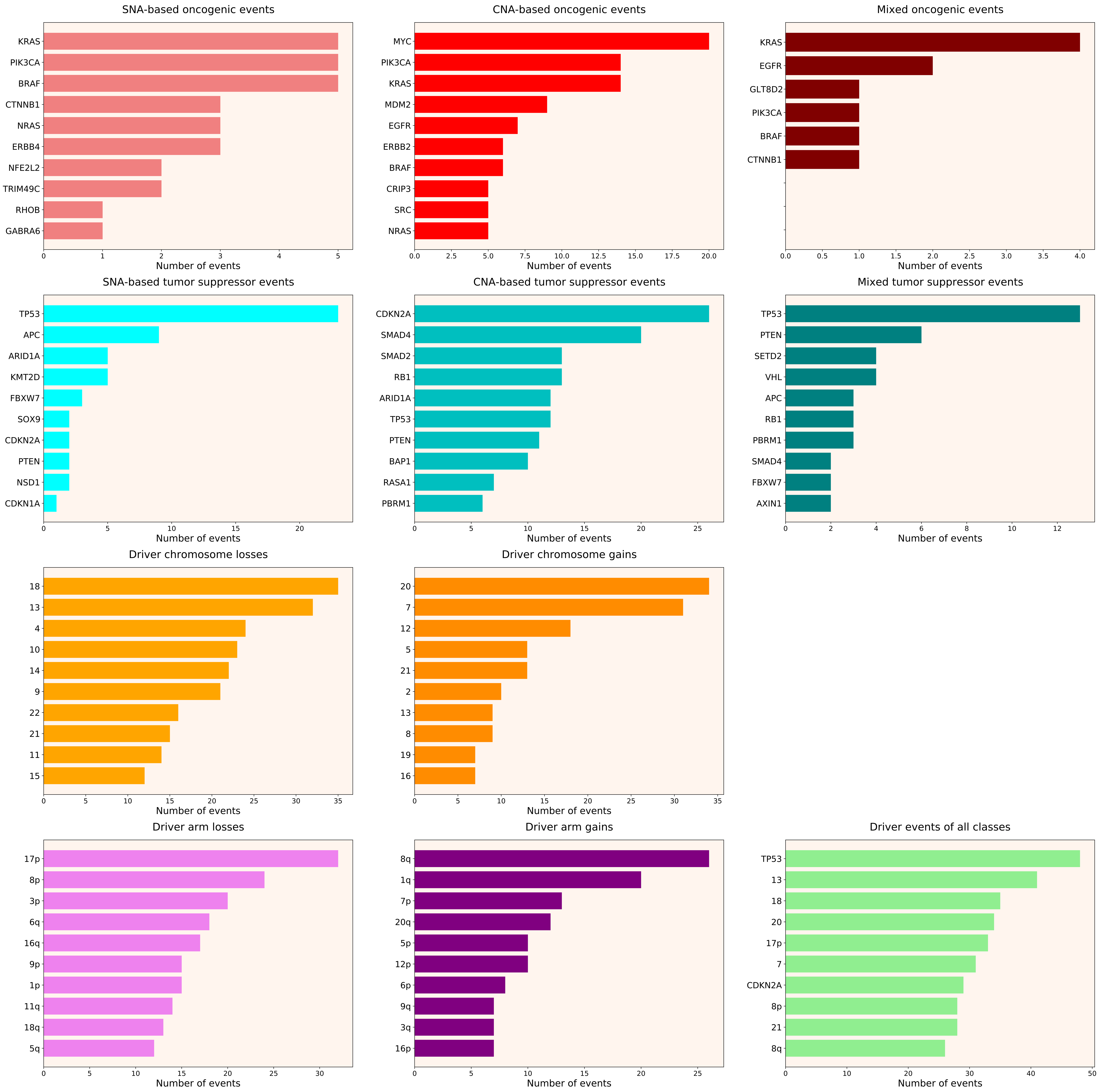

SNA-based oncogenic events
KRAS
PIK3CA
BRAF
CTNNB1
NRAS
ERBB4
NFE2L2
TRIM49C
RHOB
GABRA6
Number of events
CNA-based oncogenic events
MYC
PIK3CA
KRAS
MDM2
EGFR
ERBB2
BRAF
CRIP3
SRC
NRAS
Number of events
Mixed oncogenic events
KRAS
EGFR
GLT8D2
PIK3CA
BRAF
CTNNB1
Number of events
SNA-based tumor suppressor events
TP53
APC
ARID1A
KMT2D
FBXW7
SOX9
CDKN2A
PTEN
NSD1
CDKN1A
Number of events
CNA-based tumor suppressor events
CDKN2A
SMAD4
SMAD2
RB1
ARID1A
TP53
PTEN
BAP1
RASA1
PBRM1
Number of events
Mixed tumor suppressor events
TP53
PTEN
SETD2
VHL
APC
RB1
PBRM1
SMAD4
FBXW7
AXIN1
Number of events
Driver chromosome losses
18
13
4
10
14
9
22
21
11
15
Number of events
Driver chromosome gains
20
7
12
5
21
2
13
8
19
16
Number of events
Driver arm losses
17p
8p
3p
6q
16q
9p
1p
11q
18q
5q
Number of events
Driver arm gains
8q
1q
7p
20q
5p
12p
6p
9q
3q
16p
Number of events
Driver events of all classes
TP53
13
18
20
17p
7
CDKN2A
8p
21
8q
Number of events
